# The effect of psychosocial stimulation on the development, nutrition, and treatment outcomes of hospitalised children with severe acute malnutrition in Southern Ethiopia: a cluster randomised control trial: EPSoSAMC study

**DOI:** 10.1017/jns.2024.94

**Published:** 2025-02-11

**Authors:** Tesfalem Teshome Tessema, Andamlak Gizaw Alamdo, Eyoel B. Mekonnen, Tewodros G. Yirtaw, Fanna A. Debele, Teklu Gemechu, Tefera Belachew

**Affiliations:** 1 School of Public Health, St. Paul’s Hospital Millennium Medical College, Addis Ababa, Ethiopia; 2 SANKOFA Research and Consulting Plc, Addis Ababa, Ethiopia; 3 Jimma University College of Education, Jimma, Ethiopia; 4 Jimma University College of Public Health and Medical Sciences, Nutrition and Dietetics, Jimma, Ethiopia

**Keywords:** Development, Malnourished Children, Nutrition, Psychosocial-Stimulation, Treatment outcomes, EDHS, Ethiopia Demographic and Health Survey, FM, Fine Motor, FMoH, Federal Ministry of Health, FNP, Food and Nutrition Policy, GEE, Generalised Estimating Equations, GLM, Generalised Linear Model, GM, Gross Motor, HFIAS, Household Food Insecurity Access Scale, LA, Language, LMM, Linear Mixed Model, PS, Personal Social, SAM, Severe Acute Malnutrition, SD, Standard Deviation, SDG, Sustainable Development Goal, WAZ, Weight-for-Age -Z score, WHO, World Health Organization, WHZ, Weight for Height Z score

## Abstract

Psychosocial stimulation is one of the recommended interventions in the management of hospitalised children with severe acute malnutrition (SAM). However, there is currently limited scientific evidence supporting the effectiveness of the intervention. The study aimed to examine the effects of psychosocial stimulation on the development, nutrition, and treatment outcomes of hospitalised SAM children. A cluster-randomised controlled trial was conducted among health facilities that provide inpatient care for children with SAM in Silti Zone, Ethiopia. Fifty-eight children enrolled in the intervention facilities were provided stimulation intervention during their inpatient care and for 6 months after discharge. Sixty-eight children enrolled from control health facilities received routine inpatient care without stimulation and were followed for six months. Health education was provided to all caregivers on child health-related topics. Child development and nutrition outcomes were assessed four times using Denver II-Jimma and anthropometric measurements while the length of hospitalisation was used to measure treatment outcome. Children in the intervention group showed significantly better scores in Personal Social (p=0.001, effect size=0.77), Fine Motor (p=0.001, effect size=1.87), and Gross Motor (p=0.001, effect size=0.78) developmental domains from baseline to end line. Language domain however showed a significant difference only after discharge and intervention children scored better at six months (p<0.001, effect size=0.59). The intervention significantly improved treatment outcomes (p=0.010), but no significant changes in nutritional outcomes were documented. The findings highlighted the benefits of the intervention and the need to promote these interventions in health facilities within resource-limited settings.

## Introduction

Over the past three decades, the world has achieved significant progress in reducing child mortality. Yet, millions of children still do not live past their fifth birthday, and the burden of death is not shared equally across regions. In low-income countries, 67 children died per 1,000 live births, while only five such deaths were reported in high-income countries.^(1)^ Despite the increased attention, nutritional deficiencies remain one of the major problems contributing to child mortality.^(2)^ Globally, 149.2 million (22.0%) under-five children were stunted and 45.4 million (6.7%) were wasted and most of these children live in Africa and Asia. In Africa, 41% and 27% of children were reported wasted and stunted, respectively (UNICEF, WHO and World Bank, 2021). Based on the report of the Ethiopia Demographic and Health Survey (EDHS), 37% of under-five children were stunted, 7% wasted, and 21% underweight in the country.^(3)^ The World Health Organization (WHO) estimated that around 45% of deaths among children under 5 years of age were linked to undernutrition.^(4)^ Ethiopia has been taking substantial steps to address childhood malnutrition over the last decades and evidence of progress has also been noted nationally. The prevalence of stunting, wasting, and underweight among children under five has dropped from 44% to 37%, 10% to 7%, and 29% to 21%, respectively, between 2011 and 2019.^(3–5)^

The Ethiopia government has been implementing several initiatives to further improve childhood malnutrition in the country and meet the Sustainable Development Goal (SDG) targets particularly, SDG two and three.^(6)^ Among the key initiatives, the first Food and Nutrition Policy (FNP) was launched in 2018^(7)^ and Seqota Declaration Implementation Plan has also been carried out with the aim of ending undernutrition by 2030.^(8)^ Moreover, the Ethiopian Federal Ministry of Health (FMoH) has developed a harmonised guideline for the management of severe and moderate acute malnutrition to enhance the care and support provided to malnourished children.^(9)^ In line with the recommendation of WHO, the guidelines recommended the implementation of psychosocial stimulation interventions for children hospitalised with SAM to reduce their risk of permanent intellectual disability and emotional impairment.^(9,10)^

However, the effectiveness of psychosocial stimulation intervention for children hospitalised with SAM has been studied in few cases and inconsistent findings were also reported across key outcomes. A study conducted in Jamaica reported a significant benefit of the stimulation programme on the mental development of the children with SAM.^(11)^ Another study conducted in Bangladesh found that the intervention improved the mental development, motor development, and weight-for-age Z-score of children with SAM.^(12)^ According to a study conducted in Ethiopia, the intervention significantly improved the motor functions but not linear growth or nutritional outcomes among hospitalised children with SAM.^(13)^ However, a study conducted in Malawi reported that hospital-based psychosocial stimulation and counselling programme showed no differences in child development.^(14)^ Moreover, information contamination, high loss to follow-up, the lack of randomisation, and unequal observation between the intervention groups were some of the limitations identified in the previous studies.^(11–14)^ In Addition, the recommendations of the WHO on the implementation of psychosocial stimulation intervention were not strictly followed in all the previous studies.^(10)^ The impact of the intervention on treatment outcome indicators was not also addressed adequately in the previous studies. A systematic review conducted on psychosocial stimulation interventions for children with SAM has further indicated the need for further studies to confirm the benefit of the intervention.^(15)^ In the present study, the recommendations of the WHO on the implementation of stimulation interventions were followed. The objective of the study was to examine the effectiveness of psychosocial stimulation interventions on the development, growth, and treatment outcome children with SAM.

## Research methods and materials

### Study area, design, and sample size

**Figure 1.**
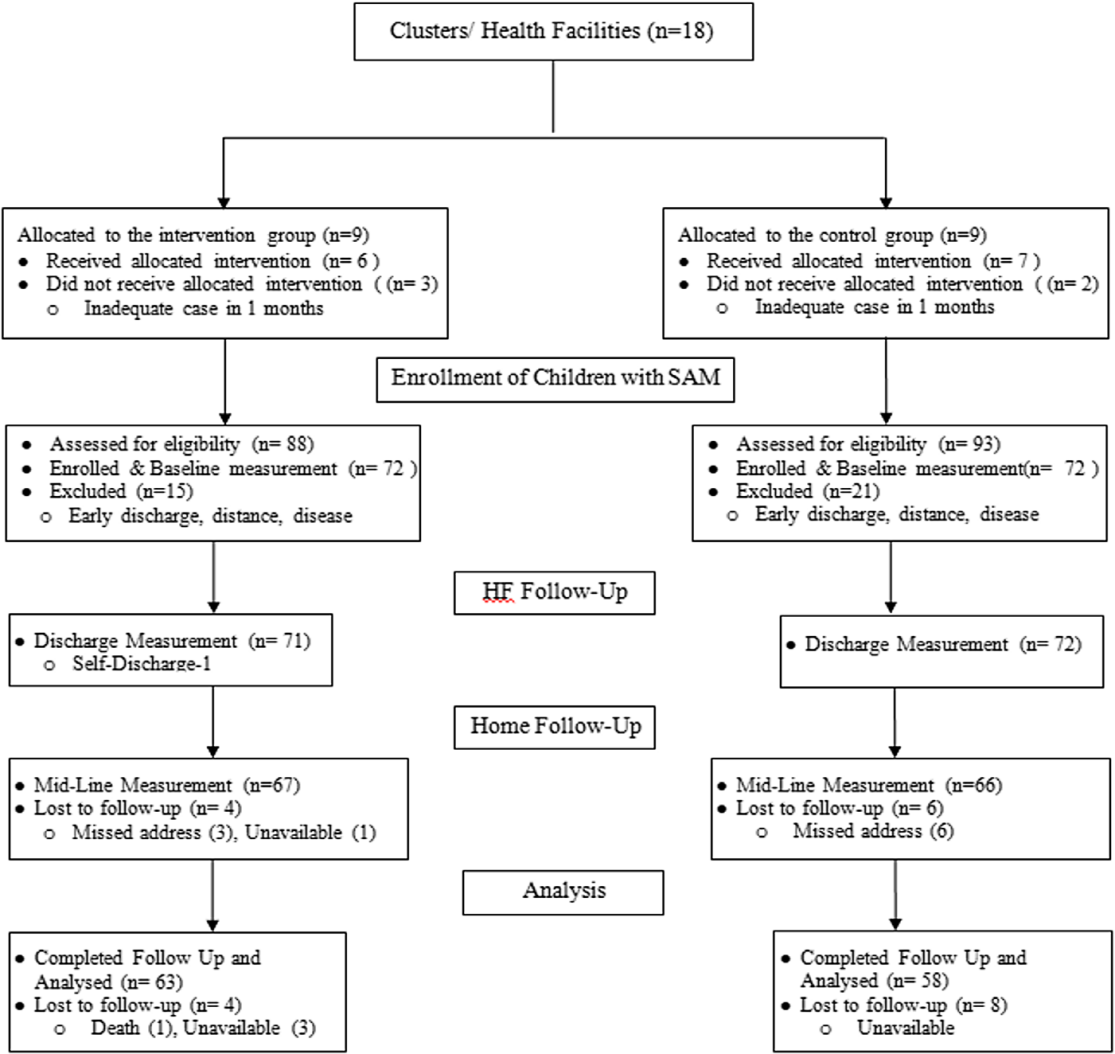
The flow chart of EPSoSAMC trail, Silti Zone, Central Ethiopia July 2023.

The study was conducted in Silti Zone, which is located in Central Ethiopia around 175 km from Addis Ababa. The study was a parallel-group cluster-randomised controlled trial that involved health facilities (clusters), which provided inpatient care for children with SAM. The trial was initially designed to enrol 18 health facilities. However, during the first one-month recruitment period, five health centres were excluded due to the absence of eligible children. The shortage of milk and the fact that the recruitment was started in the rainy season, which often affects the healthcare-seeking behaviours of the community, could have potentially contributed to the reduction in cases. Therefore, the trail included 13 health facilities (Fig. 1). Furthermore, due to the shortage of Denver Jimma II test kits, we were unable to randomly allocate the intervention across all health facilities that participated in the study. Instead, only four hospitals were randomly assigned to either the intervention or control group. To streamline the process, health centres were grouped based on their proximity to these hospitals, and they received the intervention in alignment with the allocation status of the nearby hospital.

**Figure 2.**
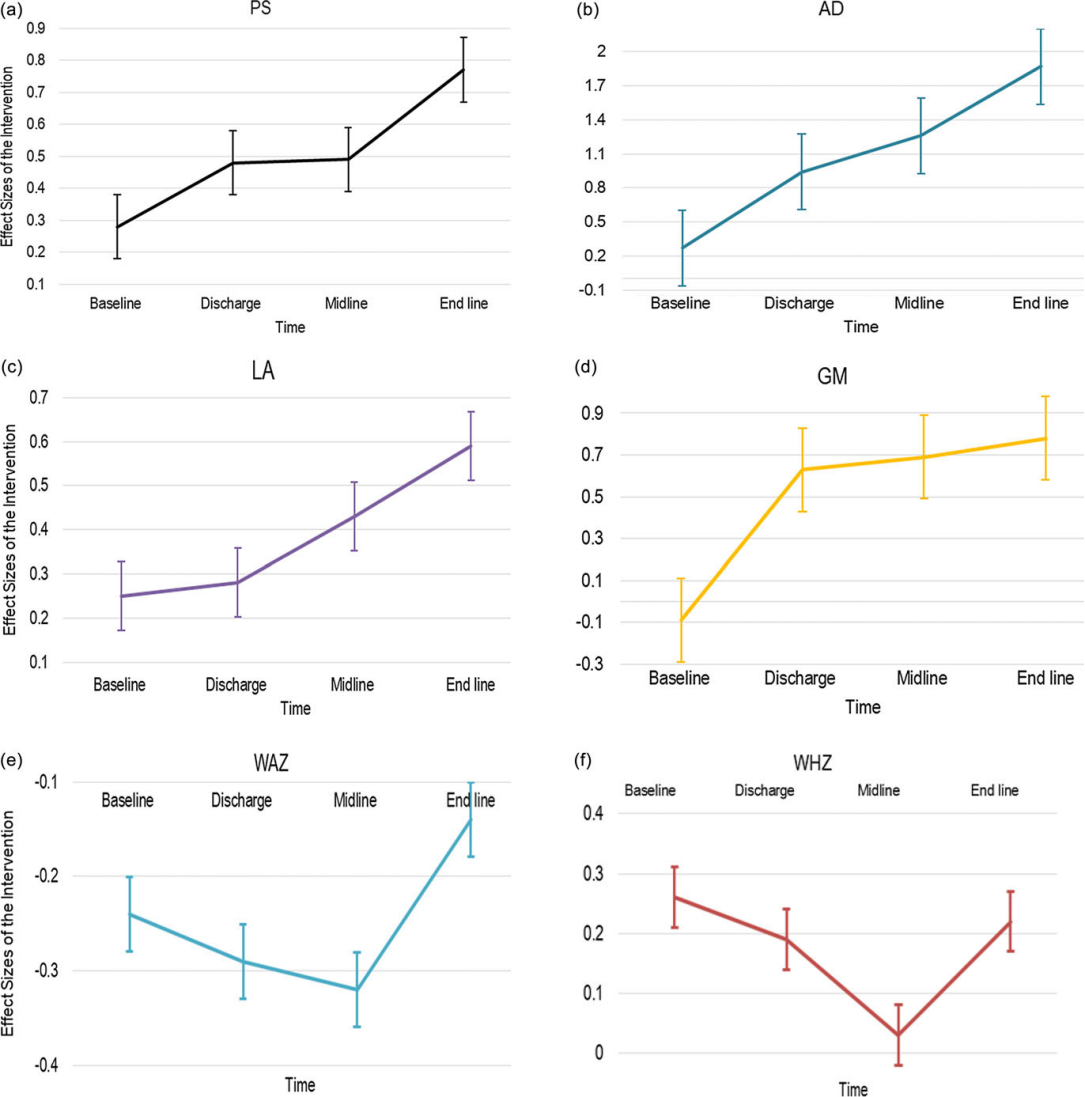
The effect size of the intervention on the development and nutritional outcomes of studied SAM children during different measurement points (Figures were based on the independent T-test), Silti Zone, Ethiopia July 2023.

The sample size was determined considering the longitudinal and clustered nature of the data. Initially, sample size based on individual randomisation was determined for comparing two means^(16)^ with the aim of detecting a 10% improvement (d=10%) from the mean (± SD) of 15.8±4.4 in fine motor development of hospitalised children with SAM.^(13)^ The number of repeated measurements (t=4), type one error (0.05), power (90%), and an assumed correlation of the repeated measures (*r*= 0.02) were considered. The sample size was further adjusted to account for the effect of clustering with an assumed Intracluster Correlation Coefficient of ρ=0.05^(17)^ and a potential dropout rate of 20%,. Therefore, a sample of 72 children with SAM aged 6 to 59 months was sufficient in each group with a total of 144 children across the two intervention arms.

### Recruitment, intervention, and follow-up

The recruitment initially planned for two months was extensively extended spanning from June 2022 to December 2022 in order to achieve the targeted sample size. During this period, trained health workers enrolled eligible children, who were transferred to the transition phase along with their caregivers. The intervention and follow-up occurred over a 13-month period, spanning from June 2022 to June 2023. In the intervention facilities, dedicated play corners were established and equipped with age and developmentally appropriate play materials. Young women, referred to as intervention workers or Play guides were also recruited from the local community. These women had completed at least secondary education and received training before the intervention. Their role was to facilitate the stimulation intervention (limited to the intervention facilities), provide health education, and conduct home visits. In the intervention facilities, children were given psychosocial stimulation interventions following the recommendations of the WHO and FMoH^(9,10,18)^; where the intervention workers facilitated a half-hour individual stimulation session daily. Moreover, children were encouraged to spend prolonged periods in the play area and caregivers were also encouraged to continue the stimulation activities. In the six-month follow-up period, intervention workers visited children five times at the end of the 1^st^ week, 2^nd^ week, 1^st^ month, 3^rd^ month, and 6^th^ month following the WHO recommendation. During each visit, intervention workers engage children in a half-hour individual stimulation session. In the control health facilities, children were given the routine inpatient care and home visits without psychosocial stimulation. All caregivers were also given counselling on health, nutrition, development, and related topics during the inpatient and follow-up periods based on health education manual used in the Ethiopian context.^(18–20)^ Further details of the trail have been presented in previous publications.^(21)^

### Data collection and outcome measurement

The primary outcome of the study was child development while the nutritional status and treatment outcome were the secondary outcomes. Testers who were trained by senior researchers who led and participated in the adaptation, standardisation and translation of the Denver II-Jimma tool administered the development tests and anthropometric measurements. They were assessed at the end of the training and all of them scored above 90%, which was agreed as an acceptable level of performance. The development and nutritional outcomes were assessed four times: at enrolment, upon discharge, and at three and six months post-discharge. The four areas of child development including Personal Social (PS), Fine Motor (FM), Language (LA), and Gross Motor (GM) were measured using the Denver II-Jimma. The tool was adapted, standardised and translated to the Ethiopian context^(22)^ and previously used in a number of studies.^(13,23,24)^ All items were tested based on the standardised test administration procedures described in the Denver II-Jimma.^(25)^ Anthropometric measurements of weight and length/height were also undertaken following the standard procedure expressed in the national protocol for the management of SAM.^(18)^ A measurement requiring greater concentration (developmental test) was administered first, followed by anthropometric measurements.^(26)^

In this study, treatment outcome was measured based on the length of stay in the nutrition unit (days). Socio-demographic and other data such as caregivers’ utilisation of key maternal health and counselling services, and the ownership of household resources were collected. Moreover, the Household Food Insecurity Access Scale (HFIAS) developed in 2007 by Food and Nutrition Technical Assistance was used to measure the level of household food insecurity.^(27)^

### Data management and analysis

A data entry operator checked, coded, and entered the collected data into EpiData Version 4.6.0.6 Software, which were transported to Stata Version 15.0 Statistical Software to manage the statistical analyses. For each development sector, a numerical outcome variable was computed based on the performance score of children. Anthropometric data of weight and height were analysed using WHO Anthro Version 3.2.2 Statistical Software and the means of the weight-for-height (WHZ) and weight-for-age (WAZ) were calculated as summary statistics representing the nutritional status of children. The length of stay in the nutrition units (days) was also used as a measure of the treatment outcome.

Independent two-sample t-test and chi-square test were used to compare the baseline characteristics between children in the control and intervention groups. The Generalized Estimating Equations (GEE) model was fitted considering each of the development and nutritional outcomes as a dependent variable. In order to account for model (correlation structure) misspecification, the models were fitted with exchangeable working correlation structure with a robust estimator, which was chosen at start and compared with other working correlation structures. The changes in the coefficient estimates and standard errors were minimal, and sometimes convergence problems were noted with the other working correlations. In addition, the robust standard errors were compared with the model-based estimators, and slight differences were seen. Therefore, the simplest structure (exchangeable) was maintained in the model-building procedure. As a balanced design, the software determined the most suitable correlation structure in the event of misspecifications. In this analysis, the interaction effect of time and treatment was examined to see if the effect of intervention has been modified with time. An independent t-test was conducted to assess the change and test the difference between the study groups with respect to the outcome variables while controlling the effect of time. It was also used in calculating the effect sizes of the intervention using the mean differences of the two groups and the Standard Deviation (SD) of the control group. Moreover, a generalized linear model (GLM) was employed to identify factors that predict the length of stay of children in the nutritional unit, which was used to measure the treatment outcome indicator. Considering the skewed nature of the time, a GLM with a normal distribution with identity link fitted on log10 transformed outcome was used to identify factors contributing to SAM children’s length of stay in the nutrition unit.

## Result

A total of 181 children with SAM (88 Intervention and 93 Control) were assessed for eligibility and 144 (72 from each of the trial arms) were enrolled in the study. Sixty-three children (87.5%) from the intervention group and 58 (80.5%) from the control group completed the six-month follow-up and were included in the analysis (Fig. 1). At baseline, there were no significant differences between the intervention and control groups in all of the background variables except the number of live children in the household (Table 1).

**Table 1.** Baseline characteristics of children and their families, Silti Zone, Central Ethiopia July 2023

		Study groups		p-v
Variables (N=121)		Intervention	Control	
The characteristics of the families/ households				
Age of mothers (Years)^†^		31.4 (5.7)	29.8 (7.0)	0.18
No of live children^†^		4.4 (2.1)	3.2 (2.2)	0.004*
Education- Mothers	Formal education	20 (31.7)	23 (39.7)	0.36
	No formal education	43 (68.3)	35 (60.3)	
Occupation- Mothers	Housewife	53 (84.1)	48 (82.8)	0.84
	Others^a^	10 (15.9)	10 (17.2)	
Education- Fathers	Primary	44 (69.8)	49 (84.5)	0.06
	Above primary	19 (30.2)	9 (15.5)	
Occupation- Fathers	Farmer	50 (79.4)	42 (72.4)	0.37
	Others^b^	13 (20.6)	16 (27.6)	
Socio-Economic status	Low	28 (44.4)	35 (60.3)	0.14
	Medium	27 (42.9)	15 (25.9)	
	High	8 (12.7)	8 (13.8)	
Food insecurity prevalence	Food security	26 (41.3)	22 (37.9)	0.71
	Food insecurity ^c^	37 (58.7)	36 (62.1)	
Key health service utilisation of the mothers				
Counselled about infant and young child feeding		55 (87.3)	49 (84.5)	0.66
FP Use		23 (36.5)	29 (50.0)	0.13
Characteristics of the children				
Age in months^†^		24.1 (7.6)	22.3 (±11.6)	0.31
Birth interval (months)^†^		25.3 (6.5)	22.8 (±9.1)	0.08
Sex	Male	30 (47.6%)	27 (46. 6%)	0.91
	Female	33 (52.4%)	31 (53.4%)	

^a^Farmer, Employed, Daily Laborer, Student, ^b^Non-Farming Business, Employed, Daily Laborer, ^c^Includes Mild, Moderate and Severe food insecurity, *p <0.05, ^†^Values are means ± SD.

### Development and nutritional status of children at baseline

When comparing the mean baseline development scores of the studied children (combined intervention and control groups) to children of the same age in the standardisation sample, significantly lower development scores were noted among the studied children across all the development domains (p<0.001). Additionally, the study found no statistically significant difference between the intervention and control groups in terms of both development and nutritional outcomes during the baseline. However, at discharge, all development domains except LA showed significant differences between the intervention and control groups. Notably, no significant differences were noted in WAZ and WHZ between the intervention and control groups at discharge (Fig. 2). Furthermore, all development domains exhibited statistically significant differences between the two groups at midline and endline measurements, although the differences in nutritional outcomes remained non-significant (Table 2).

**Table 2.** Development and nutritional scores of studied severe acute malnutrition children with the development scores of the reference population, Silti Zone, Central Ethiopia July 2023

	Sample			Baseline ^†^			Discharge ^†^			Mid line ^†^			End line ^†^		
Outcomes	Studied ^a^	Standard ^b^	p-v	Intervention	Control	p-v	Intervention	Control	p-v	Intervention	Control	p-v	Intervention	Control	p-v
PS	9.37 (3.75)	13.16 (4.54)	<0.001*	9.95 (3.10)	8.74 (4.30)	0.76	14.54 (3.70)	12.01 (5.10)	0.03*	15.46 (3.60)	13.02 (4.90)	0.003*	18.76 (3.40)	15.40 (4.30)	<0.001*
FM	12.46 (2.70)	16.35 (2.66)	<0.001*	12.86 (2.34)	12.03 (3.01)	0.95	16.63 (1.9)	14.33 (2.4)	<0.001*	18.86 (1.9)	15. 91 (2.3)	<0.001*	20.49 (2.1)	16.88 (1.9)	<0.001*
LA	12.94 (4.36)	17.27 (4.77)	<0.001*	13.55 (3.48)	12.28 (5.01)	0.11	16.43 (3.4)	15.10 (5.0)	0.08	18.79 (3.2)	16.93 (4.3)	0.009	21.33 (3.4)	18.56 (4.6)	0.008*
GM	14.10 (3.17)	19.00 (3.66)	<0.001*	13.92 (2.68)	14.24 (3.64)	0.58	17.00 (5.9)	14.14(4.5)	0.004*	19.75 (5.2)	16.90 (4.1)	0.001*	21.54 (4.7)	18.48 (3.9)	0.008*
WAZ				−2.84 (0.52)	−2.73 (0.43)	0.23	−2.63 (0.53)	−2.51 (0.43)	0.15	−2.48 (0.49)	−2.34 (0.41)	0.11	−2.10 (0.45)	−2.00 (0.38)	0.48
WHZ				−3.89 (0.42)	−4.02 (0.46)	0.14	−3.62 (0.44)	−3.72 (0.50)	0.27	−2.84 (0.47)	−2.90 (0.64)	0.83	−2.13 (0.52)	−2.30 (0.70)	0.12

PS- Personal Social, FM- Fine Motor, LA- Language, GM- Gross Motor, WAZ- Weight-for-Age -Z score, WHZ- Weight for Height Z score, SD- Standard Devision

^a^Combined(Intervention and control groups), ^b^Children of the same age in the standardisation sample, *p <0.05, ^†^Values are means ± SD.

### Effect of the intervention and factors modifying the intervention effect

Compared to children in the control group, children in the intervention group have shown consistent and significantly better score in PS (p = 0.001, effect size= 0.77), FM (p = 0.001, effect size= 1.87), and GM (p = 0.001, effect size= 0.78) from baseline to end line. However, the LA domain showed a significant difference only after discharge, and intervention children scored significantly better in the LA domain at six months (p< 0.001, effect size=0.59). For nutritional outcomes, no statistically significant changes were noted between children enrolled in the intervention and control groups (Table 3).

**Table 3.** Effect size of the intervention on the development and nutritional outcomes of studied severe acute malnutrition children, Silti Zone, Ethiopia July 2023

Outcomes	Baseline to discharge	Effect size	Baseline to midline	Effect size	Baseline to end line	Effect size
PS	2.47 (0.86, 4.08) p-value =0.003*	0.48	2.44 (0.87, 4.02) p-value =0.003*	0.49	3.37 (1.95, 4.78) p-value <0.001*	0.77
FM	2.31 (1.51, 3.11) p-value <0.001*	0.94	2.94 (2.17, 3.72) p-value <0.001*	1.26	3.61 (2.89, 4.33) p-value <0.001*	1.87
LA	1.38 (−0.18, 2.93) p-value =0.08	0.28	1.86 (0.48, 3.24) p-value =0.009*	0.43	2.72 (1.25, 4.18) p-value <0.001*	0.59
GM	2.86 (0.96, 4.76) p-value=0.004*	0.63	2.85 (1.17, 4.53) p-value= 0.001*	0.69	3.07 (1.49, 4.65) p-value <0.001*	0.78
WAZ	−0.13 (−0.30, 0.05) p-value =0.15	−0.29	−0.13 (−0.29, 0.03) p-value =0.11	−0.32	−0.05 (−0.21, 0.09) p-value =0.48	−0.14
WHZ	0.09 (−0.07, 0.27) p-value =0.27	0.19	0.02 (−0.18, 0.22) p-value =0.83	0.03	0.15 (−0.07, 0.38) p-value =0.18	0.22

PS- Personal Social, FM- Fine Motor, LA- Language, GM- Gross Motor, WAZ- Weight-for-Age -Z score, WHZ- Weight for Height Z score, *p <0.05.

The study further revealed that an increase in the age of children has also shown an increase in PS (β = 0.90, p <0.001), FM (β = 0.37, p <0.001), LA (β =0.66, p <0.001), and GM (β = 0.12, p <0.001) scores where such increases were more pronounced among younger children, particularly in the PS, FM and LA development domains. Children with better baseline development score (With small gap between the baseline and standardised score) were also found to have significantly higher PS (β = −0.25, p=0.001) and FM (β = −0.27, p=0.001) scores (Table 4). We fitted a Linear Mixed Model (LMM) using continuous outcome variables computed for developmental outcomes based on the ratio of actual pass to the expected pass for each child as a function of his/her age (The performance of a child compared to children of the same age in the standardisation sample). The result of the model however corroborated the main findings presented so far.

**Table 4.** The effect of the intervention on the development and nutritional outcome of severe acute malnutrition children and factors modifying the intervention effect, Silti Zone, Ethiopia July 2023

Variables (N=121)	Adjusted effect -PS		Adjusted effect -FM		Adjusted effect -LA		Adjusted effect –GM ^d^		Adjusted effect -WAZ		Adjusted effect -WHZ	
	β(95%CI)	p v	β(95%CI)	p v	β(95%CI)	p v	β(95%CI)	p v	β(95%CI)	p v	β(95%CI)	p v
Study group												
Intervention	−.17 (−.49, .15)	.296	.18 (−.29, .66)	.453	.13 (−.27, .54)	.521	−0.54 (−.1.32, .25)	.181	−0.14 (−.29, .02)	.089	.15 (−.01, .32)	.065
Control	Ref.		Ref.		Ref.		Ref.		Ref.		Ref.	
Time		<0.001*	<.001*	<0.001*		<0.001*		<0.001*		<0.001*		<0.001*
Discharge	3.98 (3.69, 4.27)	<.001*	2.29 (1.84, 2.75)	<0.001*	2.78 (2.37, 3.18)	<0.001*	−0.10 (−1.61, 1.40)	0.89	0.23 (0.20, 0.25)	<0.001*	0.29 (0.27, 0.32)	<0.001*
Midline	4.92 (4.64, 5.19)	<.001*	3.88 (3.45, 4.31)	<0.001*	4.66 (4.18, 5.13)	<0.001*	2.66 (1.33, 3.98)	<0.001*	0.39 (0.36, 0.42)	<0.001*	1.15 (1.08, 1.22)	<0.001*
End line	7.78 (7.44, 8.11)	<.001*	4.84 (4.32, 5.37)	<0.001*	6.29 (5.75, 6.84)	<0.001*	4.24 (2.93, 5.55)	<0.001*	0.72 (0.69, 0.76)	<0.001*	1.74 (1.64, 1.83)	<0.001*
Base line	Ref		Ref		Ref		Ref		Ref		Ref	
Group × Time		<0.001*		<0.001*		<0.001*		<0.001*		<0.001*		<0.001*
Intervn × Discharge	1.26 (0.73, 1.79)	<0.001*	1.48 (0.88, 2.09)	<0.001*	0.09 (−0.44, 0.64)	0.72	3.18 (1.10, 5.27)	0.003*	−0.02 (−0.06, z0.01)	0.13	−0.02 (−0.06, 0.02)	0.24
Intervn × Midline	1.23 (0.72, 1.75)	<0.001*	2.12 (1.54, 2.70)	<0.001*	0.58 (−0.09, 1.26)	0.09	3.17 (1.32, 5.02)	0.001*	−0.03 (−0.08, 0.02)	0.28	−0.09 (−0.19, 0.00)	0.06
Intervn × End line	2.15 (1.62, 2.69)	<0.001*	2.79 (2.12, 3.46)	<0.001*	1.44 (0.66, 2.21)	<0.001*	3.39 (1.62, 5.17)	<.001*	0.05 (−0.01, 0.11)	0.12	0.03 (−0.11, 0.18)	0.63
Intervn × Baseline	Ref		Ref		Ref		Ref		Ref		Ref	
No of live children							0.09 (−0.18, 0.37)	0.51	0.04 (0.01, 0.07)	0.02*	0.04 (0.01, 0.08)	0.02*
Food insecurity												
Food secure									−0.18 (−0.33, −0.02)	0.027*	−0.16 (−0.30, −0.01)	0.039*
Food insecure									Ref		Ref	
Linear age	0.90 (0.81, 0.99)	<0.001*	0.37 (0.25, 0.49)	<0.001*	0.66 (0.57, 0.75)	<0.001*	0.12 (0.05, 0.19)	<0.001*	−0.00 (−0.01, 0.00)	0.36	0.90 (0.81, 0.99)	<0.001*
Quadratic age	−0.01 (−0.01, −0.01)	<0.001*	−0.003 (−0.01,-0.001)	0.003*	−0.005 (−0.01,-0.003)	<0.001*	−0.01 (−0.01,0.002)	0.16	0.00 (−0.00, 0.00)	0.59	0.00 (−0.00, 0.00)	0.15
Birth interval	0.01 (−0.03, 0.01)	0.25	−0.01 (−0.03, 0.01)	0.19	−0.002 (−0.02, 0.02)	0.83	0.08 (−0.001, 0.16)	0.054	−0.01 (−0.02, 0.00)	0.17	−0.01 (−0.02, −0.01)	<0.001*
Gap ^a^	−0.25 (−0.37, −0.13)	0.001*	−0.27 (−0.43, −0.10)	0.001*	−0.09 (−0.19, 0.01)	0.067	−0.54 (−1.09, 0.02)	0.057				
Sex												
Male							1.09 (−0.14, 2.31)	0.082	−0.30 (−0.45,-0.15)	<0.001*	−0.10 (−0.25,0.05)	0.18*
Female							Ref		Ref		Ref	

PS- Personal Social, FM- Fine Motor, LA- Language, GM- Gross Motor, WAZ- Weight-for-Age -Z score, WHZ- Weight for Height Z score, ^a^Gap/ difference between the studied children and children of the same age in the standardisation sample at baseline, * *p* <0.05.

### Treatment outcome

Children in the intervention group had a mean (±SD) stay of 10.5 ± 3.1 days, whereas those in the control group stayed an average of 11.4 ± 4.3 days at the nutrition units with a statistically significant difference between the two groups (*p*=0.010). The intervention and gap in GM functions were the key predictors for the length of stay in the nutrition unit. SAM children enrolled in the intervention group have reduced length of stay in the nutrition unit compared to those in the control group (β=0.08, p=0.03). SAM child with lower GM score have also shown longer length of stay in the nutrition unit (β=0.03, p=0.019) (Table 5).

**Table 5.** Factors affecting the treatment outcome of severe acute malnutrition children, Silti Zone, Ethiopia July 2023

		Crude effect		Adjusted effect	
Variables (N=121)		β(95%CI)	p-value	β(95%CI)	p-value
Study group	Intervention	−0.02 (−0.08, 0.03)	0.42	−0.08 (−0.16, −0.01)	0.030*
	Control	Ref		Ref	
Age of mothers (years)		−0.00 (−0.01, 0.00)	0.30		
No of live children		−0.01 (−0.02, 0.00)	0.17*	−0.00 (−0.02, 0.01)	0.57
Education- Mothers	Formal education	0.03 (−0.02, 0.09)	0.25*	0.00 (−0.05, 0.08)	0.99
	No formal education	Ref		Ref	
Occupation- Mothers	Housewife	−0.08 (−0.16, −0.01)	0.030*	−0.07 (−0.15, 0.01)	0.07
	Others-farmer,	Ref		Ref	
Occupation- Fathers	farmer	−0.06 (−0.13, 0.00)	0.069*	−0.03 (−0.11, 0.04)	0.37
	Others	Ref		Ref	
Gap ^a^ GM		0.01 (−0.01, 0.03)	0.23*	0.03 (0.00, 0.05)	0.019*
Baseline WAZ		−0.04 (−0.10, 0.02)	0.16*	−0.04 (−0.09, 0.02)	0.24
Baseline WHZ		−0.05 (−0.11, 0.02)	0.17*	0.01 (−0.08, 0.09)	0.89
Sex	Male	0.04 (−0.02, 0.09)	0.15*	0.02 (−0.04, 0.08)	0.59
	Female	Ref		Ref	

GM- Gross Motor, WAZ- Weight-for-Age -Z score, WHZ- Weight for Height Z score, ^a^Gap/ difference between the studied children and children of the same age in the standardisation sample at baseline, *p-value<0.05.

## Discussion

The results of this study confirmed the benefit of the intervention in terms of improving the development outcomes of hospitalised SAM children, which was consistently reported in most of the previous studies. For instance, a longitudinal study conducted in South West Ethiopia used the same development test as this study, the Denver II-Jimma. The study reported that the intervention significantly improved the GM and FM development domains and non-significant improvement in the other domains were also documented.^(28)^ However, in this study, children in the intervention group have shown consistent and significant increase in PS, FM, and GM performances from baseline to end line. Statistically significant improvement in LA performance was also seen during home-based follow-up. Our finding revealed a much better benefit of the intervention in terms of improving the development outcomes than the one previously reported in Ethiopia. In the earlier study, SAM children receiving inpatient care at a single hospital were randomly assigned to either the intervention or control group, which could have increased the risk of information contamination. The authors also reported that the control group had access to some intervention components designed exclusively for children in the intervention group due to ethical issues. Additionally, the study experienced a high rate of loss to follow-up. These factors may have contributed to the limited likelihood of identifying substantial differences between the two groups, potentially explaining some of the disparities observed between the two studies.

A similar study conducted in Bangladesh examined the effect of adding stimulation to the routine treatment of hospitalised SAM children aged 6–24 months. Although the control group did not receive any home visits making it difficult to understand whether it is the play activities in themselves, which have had an impact or regular home visits, statistically significant benefit of the intervention in improving the mean mental and motor development score were reported.^(12)^ Another study by Grantham-McGregor, Schofield and Powell (1987) has also examined the impact of stimulation intervention on the mental development of severely malnourished children aged 6-24 months in Jamaica. It was reported that the intervention had shown a significant positive effect on the mental development of children.^(11)^ The study was, however, conducted more than three decades ago when the current standard of care for the management of SAM might have been different. On contrary, a study conducted in Malawi reported that hospital-based psychosocial stimulation and counselling programme did not contribute to improvement in developmental or nutritional outcomes.^(14)^ Unlike other studies, a 4-day hospital-based counselling and psychosocial stimulation programme were only implemented in the study with no home-based follow-up after discharge from the nutrition unit. Among others, the variations in the use of different development tests may have also contributed to the variations observed in the findings of the reviewed studies.

In this study, children with low baseline development score (higher gap between the baseline and standardised score) have shown significantly lower PS scores compared to their counter parts. A low baseline development score could create a lock-in effect, where the achievement of improved development outcome would be slower and harder than for those with a higher baseline score. Several studies have also suggested the negative impact of low baseline development score on achievement of development outcomes. For example, a cohort study of low Apgar scores and cognitive outcomes found that children who had low Apgar scores at birth (a proxy for low baseline development score) had lower IQ scores and poorer academic performance at age ten.^(29)^ Similarly, a study of centre-based childcare and differential improvements in the development of young children showed that children who had low baseline development scores benefited less from the intervention than those who had higher baseline scores.^(30)^

This study further revealed that the intervention has significant benefits in terms of improving the treatment outcome of hospitalised SAM children. Most of similar studies did not reported about the effect of stimulation intervention on the treatment outcome indicators. Contrary to our findings, a Bangladeshi study found no difference in the length of stay between the intervention and control groups. The high level of loss to follow-up and the fact that the return home were explained by the mother’s tasks at home needs to be considered in interpreting the reported finding.^(12)^ In line with a previous study conducted in Ethiopia and Malawi, the study showed no statistically significant benefit in improving the nutritional outcomes of hospitalised SAM children.^(14,28)^ However, a study conducted in Bangladesh reported the significant benefits of the intervention in terms of the nutritional outcomes where the intervention group had a higher mean WAZ than the control group.^(12)^

The study had also some limitations that need to be acknowledged. One of them was the discharge of children from nutrition units before completing the full inpatient care. As a result, we did not measure other treatment outcome indicators, such as the duration of treatment and the number of children discharged after full recovery. Another limitation was the lack of blinding of the testers. This was due to the fact that the intervention health facilities were uniquely set up with the play corners and the children were often seen in the play areas, which made it difficult to conceal the group assignment. However, we ensured the quality of testing by providing extensive theoretical and practical training to the outcome assessors.

### Conclusion

The study found the benefit of the psychosocial stimulation interventions in terms of improving the development outcomes of hospitalised SAM children in the rural areas of Ethiopia. The finding supports most of the previous studies that consistently demonstrated the benefit of stimulation intervention in improving the development outcomes. Our study has also revealed the contribution of the intervention in improving the treatment outcome of children, which has been rarely reported in the previous studies. Finally, in line with the report of majority of the studies, the stimulation interventions did not improve the nutritional outcome of children hospitalised with SAM. The study highlighted the need to promote the wider implementation of the interventions with the medico-nutritional care provided to hospitalised children with SAM. It could also call for the collaboration of key stakeholders such as the government, health facilities, Civil Society Organizations, and others to support the standardisation of the interventions for the wider implementation in the health facilities operating in resource-poor settings. Studying the long-term effects of the stimulation interventions on outcomes such as nutritional, treatment outcome, behaviours, health, academic, and other outcomes, which were inadequately addressed in this and most similar studies was also suggested.

## References

[1] UNICEF. Levels & Trends in Child Mortality: Report 2022. Published 2023. https://childmortality.org/wp-content/uploads/2023/01/UN-IGME-Child-Mortality-Report-2022.pdf (accessed September 2024).

[2] UNICEF. Unicef’s approach to scaling up nutrition [Internet]. New York; Published 2015. https://www.unicef.org/nutrition/files/Unicef_Nutrition_Strategy.pdf (accessed September 2024).

[3] EPHI, ICF. *Ethiopia Mini Demographic and Health Survey 2019: Final Report*. Rockville, Maryland, USA: EPHI and ICF; 2021.

[4] Development Initiatives. *Global Nutrition Report: Shining a Light to Spur Action on Nutrition*. Bristol, UK: Development Initiatives; 2018.

[5] CSA, ICF. Ethiopia Demographic and Health Survey 2011. Published 2011. https://dhsprogram.com/pubs/pdf/FR255/FR255.pdf (accessed August 2024).

[6] UN. Transforming our world: the 2030 Agenda for Sustainable Development Goals (SDGs). Published September 2015. https://sustainabledevelopment.un.org/content/documents/21252030%20Agenda%20for%20Sustainable%20Development%20web.pdf (accessed September 2024).

[7] Ethiopian FMoH. National Food and Nutrition Policy. Published 2018. https://www.nipn.ephi.gov.et/sites/default/files/2020-05/Food%20and%20Nutrition%20Policy.pdf (accessed July 2024).

[8] Ethiopian FMOH. Seqota declaration implementation plan (2016–2030). Int J Innov Sci Res Technol. 2020;5(7):1504–1510.

[9] Ethiopian FMoH. Guidelines for the Management of Acute Malnutrition. Addis Ababa: Ethiopia; 2016.

[10] Ashworth A , Schofield EC , Khanum S , Jackson A. *Guidelines for the Inpatient Treatment of Severely Malnourished Children*. Avenue Appia, Geneva, Switzerland: WHO; 2003.

[11] Grantham-McGregor S , Schofield W , Powell C. Development of severely malnourished children who received psychosocial stimulation: six-year follow-up. Pediatrics. 1987;79:247. 3808797

[12] Nahar B , Hamadani JD , Ahmed T , et al. Effects of psychosocial stimulation on growth and development of severely malnourished children in a nutrition unit in Bangladesh. Eur J Clin Nutr [Internet]. 2009;63(6):725–731. 10.1038/ejcn.2008.44 18772893

[13] Abessa TG , Worku BN , Wondafrash M , et al. Effect of play-based family-centered psychomotor/psychosocial stimulation on the development of severely acutely malnourished children under six in a low-income setting: a randomized controlled trial. BMC Pediatr. 2019;19(1):336. 31521161 10.1186/s12887-019-1696-z PMC6744679

[14] Daniel AI , Bwanali M , Tenthani JC , et al. A mixed-methods cluster-randomized controlled trial of a hospital-based psychosocial stimulation and counseling program for caregivers and children with severe acute malnutrition. Curr Dev Nutr. 2021;5(8):1–12. 34447897 10.1093/cdn/nzab100 PMC8382273

[15] Daniel AI , Bandsma RH , Lytvyn L , Voskuijl WP , Potani I , Van Den Heuvel M. Psychosocial stimulation interventions for children with severe acute malnutrition: a systematic review. J Glob Health. 2017;7(1):010405. 28567278 10.7189/jogh.07.010405 PMC5441448

[16] Diggle PJ , Heagerty P , Liang K-Y , Zeger SL. *Analysis of Longitudinal Data* [Internet]. Vol 13. 2^nd^ ed. New York: Oxford University Press; 1994:253 p. http://pubs.amstat.org/doi/abs/10.1198/tech.2003.s147 CN-Q. d54. 1994 519. 5/3.

[17] Hemming K , Girling AJ , Sitch AJ , Marsh J , Lilford RJ. Sample size calculations for cluster randomised controlled trials with a fixed number of clusters. BMC Med Res Methodol [Internet]. 2011;11:102. http://www.biomedcentral.com/1471-2288/11/102 21718530 10.1186/1471-2288-11-102 PMC3149598

[18] Ethiopian FMoH. Protocol for the Management of Severe Acute Malnutrition. Addis Ababa, Ethiopia: Ethiopian FMoH; 2007.

[19] Ethiopian FMoH. *Guidelines for the Management of Acute Malnutrition*. Ethiopian FMoH; 2016.

[20] Ethiopian FMoH. National Guideline on Adolescent, Maternal Infant and Young Child Nutrition. Addis Ababa, Ethiopia: Ethiopian FMoH; 2016.

[21] Tessema TT , Alamdo AG , Yirtaw TG , et al. The effects of psychosocial stimulation on the development, growth, and treatment outcome of children with severe acute malnutrition age 6–59 months in southern Ethiopia: a parallel group cluster randomized control trial (EPSoSAMC study). BMC Public Health [Internet]. 2019;19(1610):1–9. 10.1186/s12889-019-7916-5 31791303 PMC6889618

[22] Abessa TG , Worku BN , Kibebew MW , et al. Adaptation and standardization of a Western tool for assessing child development in non-Western low-income context. BMC Public Health [Internet]. 2016;16(1):1–13. 10.1186/s12889-016-3288-2 27465679 PMC4964036

[23] Worku BN , Abessa TG , Wondafrash M , et al. Effects of home-based play-assisted stimulation on developmental performances of children living in extreme poverty: a randomized single-blind controlled trial. BMC Pediatr. 2018;18(1):1–11. 29402258 10.1186/s12887-018-1023-0 PMC5800292

[24] Worku BN , Abessa TG , Wondafrash M , et al. The relationship of undernutrition/psychosocial factors and developmental outcomes of children in extreme poverty in Ethiopia. BMC Pediatr. 2018;18(1):1–9. 29426302 10.1186/s12887-018-1009-y PMC5809114

[25] Gemechu T , Nigussie B , Granitzer M , et al. Denver II-Jimma Test Items Administration Manual: Denver II-Adapted to Jimma Ethiopia. Published September 2015:1–19. https://www.researchgate.net/publication/305688899_Adaptation_and_standardization_of_a_Western_tool_for_assessing_child_development_in_non-Western_low-income_context (accessed Septmeber 2024).

[26] Yousafzai AK , Obradović J , Rasheed MA , et al. Effects of responsive stimulation and nutrition interventions on children’s development and growth at age 4 years in a disadvantaged population in Pakistan: a longitudinal follow-up of a cluster-randomised factorial effectiveness trial. Lancet Glob Heal. 2016;4(8):e548–58. 10.1016/S2214-109X(16)30100-0 27342433

[27] Coates J , Bilinsky P , Coates J. *Household Food Insecurity Access Scale (HFIAS) for Measurement of Food Access: Indicator Guide Version 3*. Washington, DC: Food and Nutrition Technical Assistance Project; 2007.

[28] Abessa TG. Play-based family-centered psychomotor/psychosocial stimulation and the recovery of severely acutely malnourished children. [dissertation on the internet). Diepenbeek (Belgium): Hasselt University; 2018. https://documentserver.uhasselt.be/handle/1942/26180 (accessed June 2024).

[29] Odd DE , Rasmussen F , Gunnell D , Lewis G , Whitelaw A. A cohort study of low Apgar scores and cognitive outcomes. Arch Dis Child Fetal Neonatal Ed. 2008;93(2):115–120. 10.1136/adc.2007.123745 PMC5141261 17916594

[30] Reynolds SA. Center-based child care and differential improvements in the child development outcomes of disadvantaged children. Child Youth Care Forum 2022;51(2):395–420. 10.1007/s10566-021-09634-0 (accessed August 2024). 35662808 PMC9162208

